# Vibegron shows high selectivity and potent agonist activity for β3-adrenoceptors, irrespective of receptor density

**DOI:** 10.1371/journal.pone.0290685

**Published:** 2023-09-01

**Authors:** Shota Yamamoto, Hotaka Kusabuka, Akane Matsuzawa, Itaru Maruyama, Takanobu Yamazaki

**Affiliations:** 1 Central Research Laboratories, Kissei Pharmaceutical Co., Ltd., Azumino, Nagano, Japan; 2 Watarase Research Center, Kyorin Pharmaceutical Co., Ltd., Nogi-machi, Tochigi, Japan; Sao Francisco University: Universidade Sao Francisco, BRAZIL

## Abstract

β3-Adrenoceptor (AR) agonists are used to treat patients with an overactive bladder (OAB). Clinical proof-of-concept data have been obtained for the β3-AR agonists vibegron, mirabegron, solabegron, and ritobegron; however, the selectivities of these agents have not been compared directly under the same experimental conditions. Moreover, the bladders of some patients express lower β3-AR densities than those of healthy individuals, and the β3-AR density might be expected to affect agonist activity. This study assessed the β3-AR selectivities of four β3-AR agonists and examined the effects of β-AR density on their pharmacological profiles. Functional cellular assays were performed using Chinese hamster ovary-K1 cells expressing three human β-AR subtypes transfected with different amounts of plasmid DNA (0.1, 0.05, 0.025 μg/well). The half-maximal effective concentration values, intrinsic activities (IAs), and β3-AR selectivities of vibegron, mirabegron, solabegron, and ritobegron were calculated to assess their pharmacological profiles. The β3-AR selectivities of vibegron, mirabegron, solabegron, and ritobegron were >7937-, 517-, 21.3-, and >124-fold higher than for β1-ARs, and >7937-, 496-, >362- and 28.1-fold higher than for β2-ARs, respectively, under the same experimental conditions. The IAs of mirabegron, solabegron, and ritobegron decreased in line with decreasing receptor density, while the IA of vibegron was maintained at the same level as that of the full agonist isoproterenol at various β3-AR densities. Vibegron has high β3-AR selectivity and exhibits full agonist activity, regardless of the β3-AR density. These results suggest that vibegron is a highly effective and safe drug for treating OAB.

## Introduction

Overactive bladder (OAB) is a symptom complex characterized by a sensation of urgency, with or without urge incontinence, usually accompanied by frequency and nocturia [1]. The medical management of OAB has traditionally relied on antimuscarinics [2]; however, although antimuscarinics have been demonstrated to improve symptoms of OAB, they also have adverse effects including dry mouth, constipation, and blurred vision, which might prompt treatment discontinuation [3]. In addition, growing evidence suggests that antimuscarinics may increase the risk of dementia in older adults [4]. β3-Adrenoceptor (AR) agonists have recently been used as a new class of therapeutic agents for OAB [5]. The efficacy of β3-AR agonists is comparable to that of antimuscarinics, but the adverse effect profile is better, and they are not thought to be associated with dementia [3,4]. β3-AR agonists are thus now used to treat OAB in many patients, including the elderly [6,7].

β-ARs are subclassified into three subtypes: β1-, β2-, and β3-ARs. In 1999, three research groups independently reported that β3-AR mRNA was expressed in human detrusor muscle [8–10], and two of these groups also revealed that β3-ARs were mainly involved in human bladder relaxation [9,10]. Several pharmaceutical companies subsequently entered their β3-AR agonists into clinical development programs for the treatment of OAB. Four β3-AR agonists, mirabegron, vibegron, solabegron, and ritobegron, underwent clinical proof-of-concept studies, and only the first two have obtained regulatory approval in several countries to date. The reasons for the success of these two drugs are unknown, but their β3-AR selectivities may be an important factor, given that β1- and β2-AR stimulation affect cardiac function [11,12]. However, although several studies have examined the β-AR selectivity of individual drugs [13–16], no studies have directly compared the β3-AR selectivities of these four drugs under the same experimental conditions.

Expression levels of β3-ARs have been reported to vary according to the pathology. For example, β3-AR mRNA expression was significantly decreased in the bladder mucosa in patients with severe bladder outlet obstruction (BOO) compared with mild BOO and controls [17]. In addition, a recent study showed that bladders of patients with urinary incontinence expressed a lower density of β3-ARs than those of healthy individuals [18]. Furthermore, the potency and efficacy of β3-AR agonists depended on the β3-AR density on the cell membrane [19]. These results suggest that the expected effects of these agents may not be achieved in patients with such pathologies. There is thus a need to assess the pharmacological profiles in relation to receptor density.

Vibegron is a novel, potent, and selective β3-AR agonist that was recently licensed for the treatment of OAB in Japan and the United States [20,21]. In the current study, we examined the pharmacological profiles of vibegron, mirabegron, solabegron, and ritobegron under the same experimental conditions, and compared the selectivities of the four drugs for each β-AR subtype. We also evaluated the effects of different β-AR densities on the pharmacological profile of each drug.

## Materials and methods

### Drugs

Vibegron was obtained from Kyorin Pharmaceutical Co., Ltd. (Tokyo, Japan), the active form of ritobegron was synthesized in our laboratory (Kissei Pharmaceutical Co., Ltd., Nagano, Japan) [22]. The following drugs were obtained from commercial sources: mirabegron (ChemScene, NJ, USA), solabegron (MedChemExpress, NJ, USA), (–)-isoprenaline (+)-bitartrate (isoproterenol) (Sigma-Aldrich, St. Louis, MO, USA), and dimethylsulfoxide (Fujifilm Wako Pure Chemical, Osaka, Japan). Each drug was dissolved in dimethylsulfoxide.

### Cell culture and transfection of human β-ARs

Chinese hamster ovary (CHO)-K1 cells (DS Pharma Biomedical Co., Ltd., Osaka, Japan) were cultured in Ham’s F-12 medium containing 10% fetal bovine serum under 5% CO_2_ at 37°C. For cAMP assay, 2×10^5^ cells/mL were inoculated onto a 96-well plate at 100 μL/well. For membrane preparation experiments, the cells were inoculated onto 150 mm dishes with the same number of cells per culture area as for the 96-well plate. The cells were cultured for about 24 h and used for β-AR transfection (see below). The DNA sequences encoding β1-AR (P08588.2), β2-AR (NP_000015.1), or β3-AR (NP_000016.1) were cloned into a pCI-Neo expression vector (Promega, WI, USA). CHO-K1 cells expressing β1-AR, β2-AR, or β3-AR were constructed by transfection with the appropriate plasmid DNA (β1-, β2-, or β3-AR) using Lipofectamine 2000 (Life Technologies Japan Ltd., Tokyo, Japan), according to the manufacturer’s instructions. Human β-AR plasmid DNA was diluted with Opti-MEM (Life Technologies Japan Ltd.) (0.1 μg plasmid DNA/25 μL Opti-MEM) for each subtype. Lipofectamine was diluted with Opti-MEM (0.5 μL Lipofectamine/25 μL Opti-MEM) and incubated for 5 min at room temperature. Solutions containing plasmid DNA and Lipofectamine, respectively, were mixed in equal amounts and incubated for 20 min at room temperature. The mixture (50 μL/well or 23.735 mL/dish) was added to the above CHO-K1 cells inoculated onto a 96-well plate or 150 mm dish. The transfected CHO-K1 cells were then cultured for about 24 h and used for the following experiments. Similar experiments were performed after reducing the amounts of transfected plasmid DNA to 0.05 and 0.025 μg.

### cAMP assay

Cells that had been cultured for about 24 h after transfection were subjected to cAMP assay. The cells were incubated with vibegron, mirabegron, solabegron, ritobegron, or isoproterenol for 30 min at 37°C in the presence of 0.5 mM 3-isobutyl-1-methylxanthine, and intracellular cAMP accumulation was evaluated using a cAMP Gs Dynamic kit (PerkinElmer, MA, USA). Fluorescence emissions at 665 nm and 620 nm were detected using a PHERAstar FSX plate reader (BMG-Labtech Japan Ltd., Saitama, Japan).

### Membrane preparations

At 24 h post-transfection, cells in 150 mm dishes were washed with Dulbecco’s phosphate-buffered saline and detached with a cell scraper into 10 mM HEPES buffer (pH 7.4) containing 154 mM NaCl, 0.7 mM EDTA-2Na, and protease inhibitor cocktail (Nacalai Tesque, Kyoto, Japan) (membrane preparation buffer). The buffer was then removed by centrifugation at 1880 ×*g* for 10 min, the cells were resuspended in membrane preparation buffer, and centrifuged at 1880 ×*g* for 10 min. After removing the buffer, the cells were resuspended in homogenate buffer (6.6 mM HEPES, 102.7 mM NaCl, 3.3 mM NaHCO_3_, 2.1 mM EDTA-2Na, and protease inhibitor cocktail, pH 7.4) and frozen at −80°C for 1 h. The cells were thawed and homogenized using an ultrasonic homogenizer. Nuclei were removed by centrifugation at 1310 ×*g* for 10 min, and the membrane fraction was centrifuged at 92,691 ×*g* for 60 min at 4°C. The pellet was resuspended in 10 mM HEPES buffer (pH 7.4) containing 0.1 mM EDTA-2Na and stored at −80°C until use. Protein concentration was determined using a Pierce BCA Protein Assay kit (Thermo Fisher Scientific, Waltham, MA, USA).

### Radioligand-binding assay

Aliquots of membrane preparations resuspended in binding buffer (20 mM HEPES, 100 mM NaCl, and 10 mM MgCl_2_, pH 7.4) were incubated with [^125^I]-iodocyanopindolol (ICYP) (PerkinElmer) for 90 min in a final volume of 200 μL at 27°C for each β-AR. β1-, β2-, and β3-AR saturation binding assays were carried out using [^125^I]-ICYP ligands, ranging from 16–1000, 16–1000, and 31–2000 pM, respectively. Nonspecific binding was determined in the presence of 10 μM alprenolol for β1- and β2-ARs and 100 μM SR59230A for β3-ARs. After incubation, the membranes were filtered onto a 96-well white microplate with bonded GF/B filter preincubated in 0.3% polyethylenimine for 30 min and washed four times with ice-cold wash buffer (50 mM Tris-HCl buffer, pH 7.4). Radioactivity on the filter was counted using a MicroBeta2 microplate counter (PerkinElmer) with quenching correction, after the addition of 100 μL of Microscint 20 (PerkinElmer) and shaking for 5 min. Saturation experiments were analyzed by GraphPad Prism 6.0 (GraphPad software, San Diego, CA, USA) using the rectangular hyperbolic equation derived from the equation of a bimolecular reaction and the law of mass action: B = (B_max_ × [F]) / (K_D_ + [F]), where B is the amount of ligand bound at equilibrium, B_max_ is the maximum number of binding sites, [F] is the concentration of free ligand, and K_D_ is the ligand dissociation constant.

### Data analysis

In the cAMP assay, the half-maximal effective concentration (EC_50_) value was calculated for each agonist based on its concentration–response curve using GraphPad Prism 6.0 nonlinear regression analysis. The intrinsic activity (IA) of each drug was calculated as the proportion of the maximum response to 1×10^−5^ M when the maximum response to isoproterenol was taken as 1.00. When the IA of the drug was >0.5, an EC_50_ value was calculated. β3-AR selectivity was calculated by comparing the EC_50_ values. The concentration of each drug required to produce a 50% maximal response induced by isoproterenol (Iso_50_) was calculated to assess the potency of the drugs.

## Results

### EC_50_, IA, and β3-AR selectivity for each β3 agonist

**Fig 1:**
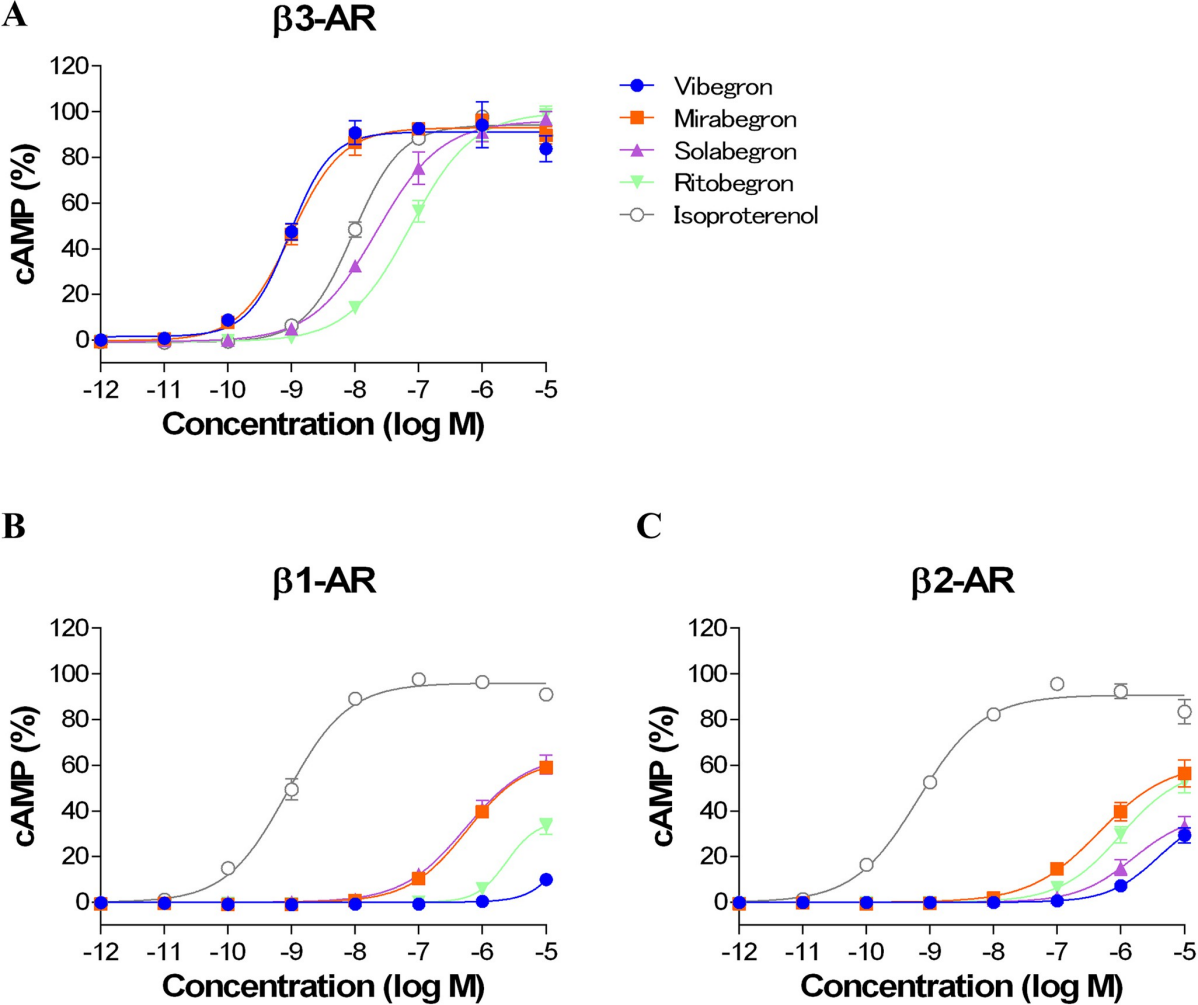
Effects of vibegron, mirabegron, solabegron, ritobegron, and isoproterenol on cAMP accumulation in CHO-K1 cells transfected with 0.1 μg/well plasmid DNA for human β3-adrenoceptor (AR) (A), β1-AR (B), or β2-AR (C). Each cAMP level expressed as mean ± standard error (SEM), taking the maximum response to isoproterenol as 100%. Each point expressed as mean ± SEM of four (vibegron, mirabegron, solabegron, and ritobegron) or eight (isoproterenol) experiments.

Cells transfected with 0.1 μg/well, which allowed sufficient evaluation of the agonistic activities of the drugs for each β-AR, were used in the experiments. All four drugs (vibegron, mirabegron, solabegron, and ritobegron) induced a concentration-dependent increase in the accumulation of cAMP in cells expressing β3-ARs (Fig 1A), with EC_50_ values of 1.26, 1.15, 27.6, and 80.8 nM and IAs of 0.93, 0.94, 0.96, and 0.99, respectively (Table 1). In cells expressing β1- and β2-ARs, the EC_50_ value for vibegron was >10 μM. Mirabegron and solabegron also had agonist activities in cells expressing β1-ARs, with EC_50_ values of 594 and 588 nM, respectively, while mirabegron and ritobegron had agonist activities in cells expressing β2-ARs, with EC_50_ values of 570 and 2273 nM, respectively (Fig 1B and 1C and Table 1). The β3-AR selectivities of vibegron, mirabegron, solabegron, and ritobegron were thus >7937-, 517-, 21.3-, and >124-fold higher than for β1-ARs, and >7937-, 496-, >362-, and 28.1-fold higher than for β2-ARs under the same experimental conditions (Table 1). Vibegron thus had the highest β3 selectivity among the four drugs.

**Table 1:** Effects of vibegron, mirabegron, solabegron, ritobegron, and isoproterenol on cAMP accumulation and β3-adrenoceptor (AR) selectivities in CHO-K1 cells expressing human β-ARs.

Drug	EC_50_ (nM) (Intrinsic activity)			β3-AR selectivity	
	β1-AR	β2-AR	β3-AR	vs β1-AR	vs β2-AR
Vibegron	>10,000	>10,000	1.26 ± 0.40	>7937	>7937
	(0.10 ± 0.01)	(0.29 ± 0.03)	(0.93 ± 0.05)		
Mirabegron	594 ± 122	570 ± 235	1.15 ± 0.24	517	496
	(0.59 ± 0.02)	(0.56 ± 0.06)	(0.94 ± 0.02)		
Solabegron	588 ± 105	>10,000	27.6 ± 9.08	21.3	>362
	(0.60 ± 0.04)	(0.33 ± 0.04)	(0.96 ± 0.03)		
Ritobegron	>10,000	2273 ± 1339	80.8 ± 12.6	>124	28.1
	(0.33 ± 0.03)	(0.53 ± 0.05)	(0.99 ± 0.02)		
Isoproterenol	0.89 ± 0.14	0.78 ± 0.16	10.7 ± 1.67	0.08	0.07
	(0.95 ± 0.01)	(0.91 ± 0.02)	(0.95 ± 0.01)		

Data presented as mean ± standard error of four (vibegron, mirabegron, solabegron, and ritobegron) or eight (isoproterenol) experiments. Intrinsic activity calculated with cAMP accumulation as 1.00 in maximal response of isoproterenol for each β-AR. β3-AR selectivity calculated by comparison between half-maximal effective concentration (EC_50_) values. CHO-K1 cells were transfected with 0.1 μg/well plasmid DNA for human β-ARs.

### β-AR agonist activity profiles of drugs in cells with different receptor densities

The densities of β-ARs expressed on the cell membrane were evaluated by radioligand-binding assay. The B_max_ values in cells transfected with 0.1, 0.05, and 0.025 μg/well were 781, 245, and 153 fmol/mg for β1-ARs, 713, 231, and 108 fmol/mg for β2-ARs, and 347, 222, and 116 fmol/mg for β3-ARs, respectively (Table 2), and these decreased as the amount of transfected β-AR plasmid DNA decreased in all β-AR subtypes.

**Table 2:** B_max_ values of [^125^I]-iodocyanopindolol binding to human β1-, β2-, and β3-adrenoceptor (AR) sites on CHO-K1 cells transfected with each β-AR plasmid DNA.

Amount of plasmid DNA (μg/well)	B_max_ (fmol/mg)		
	β1-AR	β2-AR	β3-AR
0.1	781 ± 24	713 ± 41	347 ± 25
0.05	245 ± 13	231 ± 3	222 ± 11
0.025	153 ± 7	108 ± 5	116 ± 22

Data presented as mean ± standard error of three experiments. B_max_, maximum number of binding sites.

**Fig 2:**
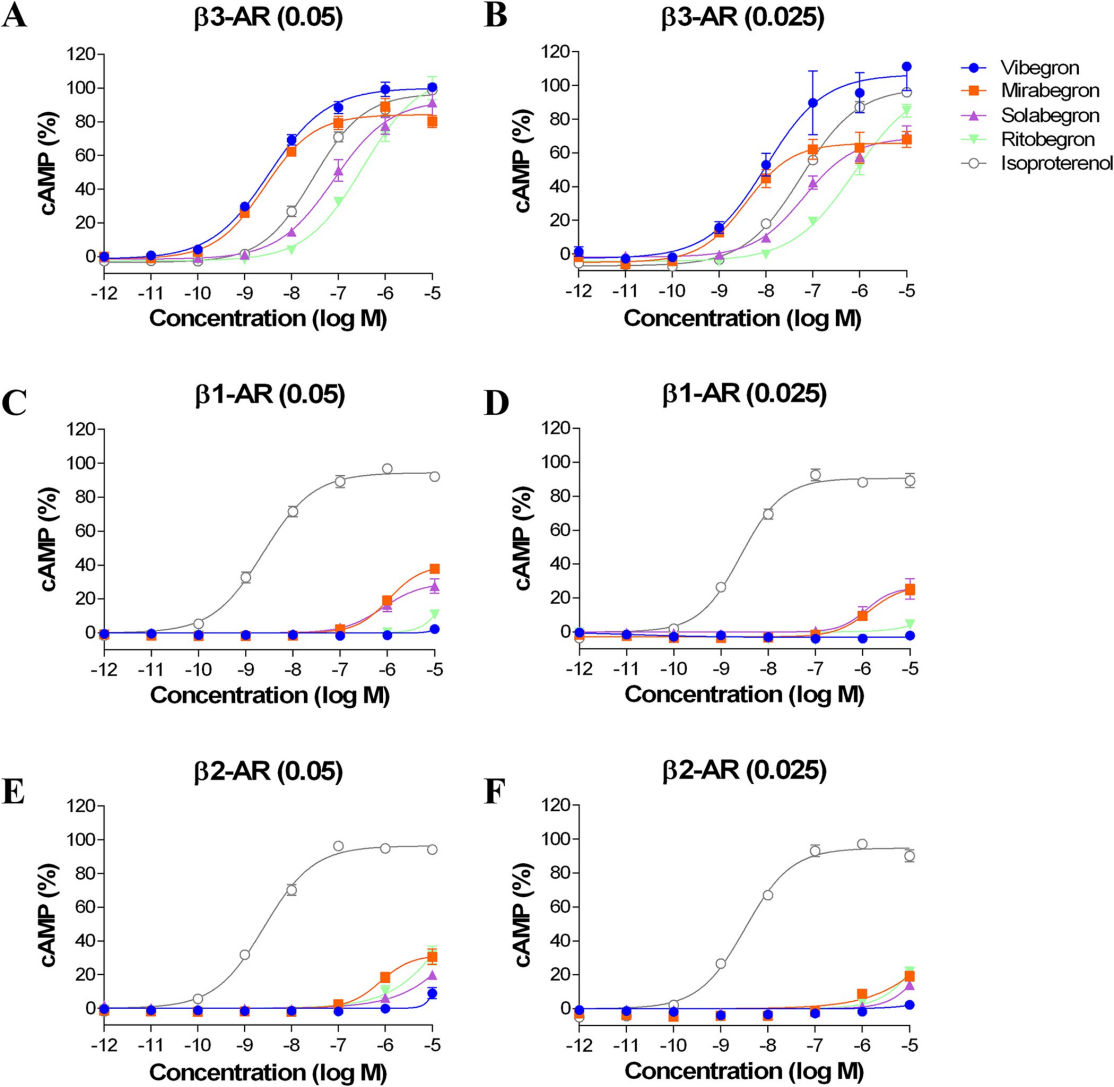
Effects of vibegron, mirabegron, solabegron, ritobegron, and isoproterenol on cAMP accumulation in CHO-K1 cells transfected with 0.05 μg/well plasmid DNA (A, C, E) and 0.025 μg/well plasmid DNA (B, D, F) for each β-adrenoceptor (AR) subtype. Each cAMP level expressed as mean ± standard error (SEM), taking the maximum response to isoproterenol as 100%. Each point expressed as mean ± SEM of four (vibegron, mirabegron, solabegron, and ritobegron) or eight (isoproterenol) experiments.

In cells expressing β3-ARs, the EC_50_ and IA of each drug were compared between cells transfected with 0.05 and 0.025 μg/well. As observed in the cAMP assay, the concentration–response curves were shifted to the right or below and the EC_50_ values increased as the amount of plasmid DNA decreased, except for solabegron (Table 3 and Fig 2A and 2B). The IAs of vibegron in cells transfected with 0.05 and 0.025 μg/well were 1.00 and 1.07, respectively, and these were maintained at the same level as that of isoproterenol, regardless of the receptor density. In contrast, the IAs of mirabegron, solabegron, and ritobegron were 0.84, 0.91, and 1.00 in cells transfected with 0.05 μg/well, and 0.66, 0.68, and 0.85 in cells transfected with 0.025 μg/well, respectively, and all decreased with decreasing receptor density (Table 3).

**Table 3:** Effects of vibegron, mirabegron, solabegron, ritobegron, and isoproterenol on cAMP accumulation in CHO-K1 cells expressing human β-adrenoceptors (ARs) under different plasmid DNA transfection (TF) conditions.

Drug	EC_50_ (nM) (Intrinsic activity)					
	TF 0.05 μg/well			TF 0.025 μg/well		
	β1-AR	β2-AR	β3-AR	β1-AR	β2-AR	β3-AR
Vibegron	>10,000	>10,000	3.83 ± 0.93	>10,000	>10,000	12.5 ± 2.41
	(0.02 ± 0.01)	(0.09 ± 0.03)	(1.00 ± 0.03)	(0.00 ± 0.00)	(0.03 ± 0.01)	(1.07 ± 0.14)
Mirabegron	>10,000	>10,000	2.81 ± 0.60	>10,000	>10,000	4.17 ± 0.89
	(0.38 ± 0.02)	(0.31 ± 0.05)	(0.84 ± 0.04)	(0.25 ± 0.03)	(0.20 ± 0.03)	(0.66 ± 0.06)
Solabegron	>10,000	>10,000	85.1 ± 5.84	>10,000	>10,000	83.6 ± 29.0
	(0.28 ± 0.04)	(0.20 ± 0.02)	(0.91 ± 0.10)	(0.25 ± 0.06)	(0.14 ± 0.03)	(0.68 ± 0.05)
Ritobegron	>10,000	>10,000	398 ± 104	>10,000	>10,000	1523 ± 950
	(0.11 ± 0.02)	(0.32 ± 0.05)	(1.00 ± 0.07)	(0.04 ± 0.01)	(0.22 ± 0.03)	(0.85 ± 0.04)
Isoproterenol	2.39 ± 0.21	2.93 ± 0.52	34.0 ± 6.87	3.21 ± 0.54	3.64 ± 0.49	70.3 ± 14.2
	(0.94 ± 0.01)	(0.97 ± 0.01)	(0.96 ± 0.02)	(0.91 ± 0.01)	(0.95 ± 0.02)	(0.97 ± 0.01)

Data presented as mean ± standard error of four (vibegron, mirabegron, solabegron, and ritobegron) or eight (isoproterenol) experiments. Intrinsic activity calculated with cAMP accumulation taken as 1.00 in maximal response of isoproterenol for each β-AR. EC_50_, half-maximal effective concentration.

In cells expressing β1- and β2-ARs, the EC_50_ values of all four drugs were >10 μM (Table 3 and Fig 2C–2F). The IAs of all drugs decreased in cells transfected with 0.025 μg/well compared with cells transfected with 0.05 μg/well (Table 3).

### Potency of each β3-AR agonist

The EC_50_ values of vibegron, mirabegron, solabegron, and ritobegron in β3-AR-expressing cells transfected with 0.025 μg/well were 12.5, 4.17, 83.6, and 1523 nM, respectively (Table 3). However, the EC_50_ indicates the concentration that produces a response of 50% of the E_max_ for that drug, and in the case of partial agonists, the EC_50_ was lower than the 50% of E_max_ response for isoproterenol (Fig 2). We therefore calculated the Iso_50_ to assess the potency of each drug for β3-ARs. The rank of Iso_50_ value was 22.5 nM (vibegron) < 26.9 nM (mirabegron) < 252 nM (solabegron) < 943 nM (ritobegron) in β3-AR-expressing cells transfected with 0.025 μg/well, with similar trends for the other transfection conditions (Table 4).

**Table 4:** Potency of β3-adrenoceptor (AR) agonists assessed by cAMP accumulation in CHO-K1 cells expressing human β3-AR under different plasmid DNA transfection (TF) conditions.

Drug	Iso_50_ (nM)		
	TF 0.1 μg/well	TF 0.05 μg/well	TF 0.025 μg/well
	347 fmol/mg	222 fmol/mg	116 fmol/mg
Vibegron	1.26 ± 0.29	3.74 ± 0.73	22.5 ± 14.5
Mirabegron	1.32 ± 0.26	4.45 ± 0.66	26.9 ± 7.51
Solabegron	31.0 ± 10.8	119 ± 27.1	252 ± 78.4
Ritobegron	81.5 ± 15.4	303 ± 60.7	943 ± 244
Isoproterenol	11.8 ± 1.78	37.9 ± 6.40	78.0 ± 10.9

Data presented as mean ± standard error of four (vibegron, mirabegron, solabegron, and ritobegron) or eight (isoproterenol) experiments. Iso_50_, the concentration of each drug required to produce a 50% of the maximal response induced by isoproterenol.

## Discussion

In the present study, we evaluated the agonist activity of the β3-AR agonists vibegron, mirabegron, solabegron, and ritobegron, developed for the treatment of OAB, against each β-AR subtype (β1-, β2-, and β3-ARs), using functional assays indexed to cAMP accumulation, and compared their β3-AR selectivities. Differences in β3-AR selectivity were observed among the four drugs, in the order vibegron > mirabegron > solabegron = ritobegron. Vibegron showed the highest β3-AR selectivity, which was >7937-fold higher than for β1- and β2-ARs. Mirabegron showed similar β3-AR agonist activity to vibegron, but it also demonstrated agonist activity for β1- and β2-ARs (EC_50_ = 594 and 570 nM), with β3-AR selectivities of 517-fold for β1-ARs and 496-fold for β2-ARs. To date, no studies have clearly demonstrated the direct agonist activity of mirabegron in β1- and β2-ARs, although it has shown affinities for β1-and β2-ARs in binding assays, with inhibition constants (Ki) values of 383 and 977 nM, respectively [23]. The current functional assay results may reflect the binding affinity of mirabegron for each β-AR subtype. Previous studies have reported the β3-AR selectivity of each drug individually [13–16], but experimental conditions have differed among the studies, making it difficult to compare the selectivities of the four drugs. In the current study, we compared the drugs under identical conditions, and showed that vibegron and mirabegron had higher β3-AR selectivities than solabegron and ritobegron.

Both β1- and β2-ARs are expressed in the heart, and drugs with poor β3-AR selectivity may thus produce cardiovascular side effects, such as increased heart rate and force of contraction [11,12]. Drugs with weaker effects on β1- and β2-ARs, at doses resulting in adequate agonist activity on β3-ARs, may thus be more desirable for the treatment of OAB. The two drugs currently used in clinical practice, vibegron and mirabegron, have high selectivity for β3-ARs and are thus therefore considered to be safe.

The β3-AR density in the urinary bladder has been reported to differ according to the disease pathology [18], and the agonist activities of the compounds may thus differ according to the receptor density on the plasma membrane [19]. We therefore examined the effect of β-AR density on the plasma membrane on the pharmacological profile of each drug. In β1- or β2-AR-expressing cells, the IAs of all four drugs decreased with decreasing receptor density. In contrast, in β3-AR-expressing cells, the IAs for mirabegron, solabegron, and ritobegron decreased with decreasing receptor density, but no such change occurred for vibegron. The detailed reasons for the IA decreases with the former three agonists are unknown. However, agonists with potency emanating from high efficacy are called efficacy-dominant agonists, while other agonists with potency emanating from high affinity and concomitant low efficacy are called affinity-dominant agonists [24]. The maximal responses to efficacy-dominant agonists are more resistant to decreases in receptor density than the responses to affinity-dominant agonists [24]. Vibegron may thus be classified as efficacy-dominant agonists. In a recent study comparing the agonist activity profiles of vibegron and mirabegron, the respective IAs (E_max_) differed between 99.2% and 80.4%, respectively [25]. The current results also showed differences in the IAs of vibegron and mirabegron depending on the receptor density. It is therefore important to consider β3-AR densities approximating those in human bladders when comparing the pharmacological profiles of drugs.

Significant reductions in β3-AR mRNA expression have been reported in the bladders of subjects with severe BOO compared with mild BOO and healthy individuals [17]. In addition, the β3-AR density in the bladder in healthy individuals was reported to be 155 fmol/mg, compared with 100 fmol/mg in patients with incontinence [18]. In the present study, β3-AR densities in cells transfected with 0.025 μg/well (B_max_ 116 fmol/mg) approximated the density in the urinary bladder in incontinent patients. Under these conditions, vibegron showed full agonist activity (IA = 1.07) and ritobegron showed strong agonist activity (IA = 0.85), but mirabegron and solabegron showed only partial agonist activity (IA = 0.66, 0.68, respectively). Notably, there were clear differences in the IAs of vibegron and mirabegron, although both drugs are used in clinical practice.

Based on the maximum (C_max_) and minimum observed plasma concentrations (C_min_) of once-daily 50 mg vibegron and mirabegron [26,27], as the recommended Japanese clinical doses, the C_max,u_ and C_min,u_ for the unbound forms, taking into account the plasma-protein-binding rate of each drug, were calculated as 56 nM and 14 nM for vibegron and 20 nM and 3 nM for mirabegron, respectively. Applying these values to the dose-response curve of β3-AR-expressing cells transfected with 0.025 μg/well, the cAMP activity of vibegron was calculated to be about 82% in C_max,u_ and 59% in C_min,u_, while the cAMP activity of mirabegron was about 53% in C_max,u_ and decreased to about 27% in C_min,u_. These findings indicate that vibegron maintained strong β3-AR agonist activity compared with mirabegron, which could affect the relative efficacies of the two drugs in clinical practice, with vibegron acting more strongly and continuously in bladder tissues expressed β3-ARs.

The desensitization of receptors is an important factor that negatively affects drug efficacy. Compared with β2-AR, the β3-AR has been reported to be less susceptible to desensitization because it lacks the C-terminal phosphorylation site involved in desensitization [28]. In addition, β2- and β3-AR are involved in bladder relaxation in rats [29]. Following pretreatment of isolated rat bladder strips with fenoterol, a β2-AR selective agonist, and subsequent washout, marked attenuation of the bladder relaxant effect was observed after fresh addition of the drug, whereas the β3-AR agonist mirabegron did not produce such attenuation [30]. Although this study did not evaluate the desensitization of β3-AR by vibegron, in a 52-week long-term efficacy study, attenuation of the efficacy of 50 mg (therapeutic dose) and 100 mg (supratherapeutic dose) of vibegron was not observed [31]. Therefore, the possibility of desensitization of β3-AR by vibegron is considered to be very low.

β1- and β2-AR densities vary in the regions of the human heart, with β1- and β2-AR densities in pacemaker cells in the sinoatrial node, which control the heartbeat, being 4.2- and 2.6-fold higher, respectively, compared with the atrium [32]. The β1- and β2-AR densities in the human right atrium have been reported to be about 54 and 22 fmol/mg [11], and the densities in the human sinoatrial node are estimated to be about 227 fmol/mg and 57 fmol/mg, respectively. In the current study, the β1-AR density in cells transfected with 0.05 μg/well (245 fmol/mg) was close to the estimated density in the human sinoatrial node, but vibegron showed no β1-AR agonist activity in these cells. The lowest β2-AR density in β2-AR-expressing cells was 108 fmol/mg, which was higher than the estimated density in the human sinoatrial node, but vibegron also showed no β2-AR agonist activity in these cells, suggesting that vibegron had no β2-AR agonist activity in cells with receptors at densities similar to those in the human sinoatrial node. These results suggest that vibegron is unlikely to affect the heart rate via β1- and β2-ARs expressed in the sinoatrial node. Indeed, it has been reported that vibegron does not affect the incidence of increased blood pressure and hypertension at therapeutic doses in clinical trials [33,34]. Additionally, in a 52-week long-term clinical trial, a supratherapeutic dose (100 mg) of vibegron did not affect vital signs (systolic blood pressure, diastolic blood pressure, and pulse) [31], and our results support this clinical evidence.

## Conclusions

In this study, we evaluated the activities of the β3-AR agonists vibegron, mirabegron, solabegron, and ritobegron on β-AR subtypes under the same experimental conditions, and determined the effects of receptor density on the pharmacological profiles of the drugs. Vibegron showed the highest β3-AR selectivity and demonstrated full agonist activity, regardless of the β3-AR density. These results suggest that vibegron is a highly effective and safe drug for the treatment of OAB.
